# Base excision repair in chromatin: A tug-of-war for DNA damage

**DOI:** 10.1016/j.dnarep.2025.103908

**Published:** 2025-10-23

**Authors:** Abigayle F. Vito, Daniel J. Boesch, Ava M. Hammons, Bret D. Freudenthal, Tyler M. Weaver

**Affiliations:** a Department of Biochemistry and Molecular Biology, University of Kansas Medical Center, Kansas City, Kansas 66160, USA; b Department of Cancer Biology, University of Kansas Medical Center, Kansas City, Kansas 66160, USA; c University of Kansas Cancer Center, Kansas City, Kansas 66160, USA; d Department of Biochemistry and Molecular Genetics, University of Virginia School of Medicine, Charlottesville, Virginia 22908, USA; e University of Virginia Comprehensive Cancer Center, Charlottesville, Virginia 22908, USA

**Keywords:** Base Excision Repair, DNA damage, Chromatin, Nucleosome, DNA sculpting

## Abstract

Base excision repair (BER) is a genome surveillance pathway responsible for repairing DNA base lesions distributed throughout the chromatinized eukaryotic genome. However, chromatin structure acts as a dynamic structural barrier that restricts access to DNA and must be overcome for BER to proceed efficiently. In this perspective, we summarize recent advances that have shaped our understanding of BER in chromatin, with a focus on the structural mechanisms employed by core BER enzymes to recognize and repair DNA lesions within the nucleosome. We highlight how DNA accessibility dictates BER enzyme activity and discuss the concepts of localized and global DNA sculpting as emerging strategies for lesion recognition and repair. We propose that BER within the nucleosome represents a molecular “tug-of-war”, where the histone octamer and the BER enzymes are in a constant competition for access to the damaged nucleosomal DNA. The outcome of this competition is dictated by the position of the DNA lesion within the nucleosome, which ultimately defines the efficiency of BER enzymes within chromatin. We also explore possible mechanisms used by ATP-dependent chromatin remodeling to facilitate BER within the nucleosome. Together, these recent advances provide a framework for understanding BER in chromatin and outline key unanswered questions regarding chromatin-based BER.

## 1. Introduction

**Fig. 1.**
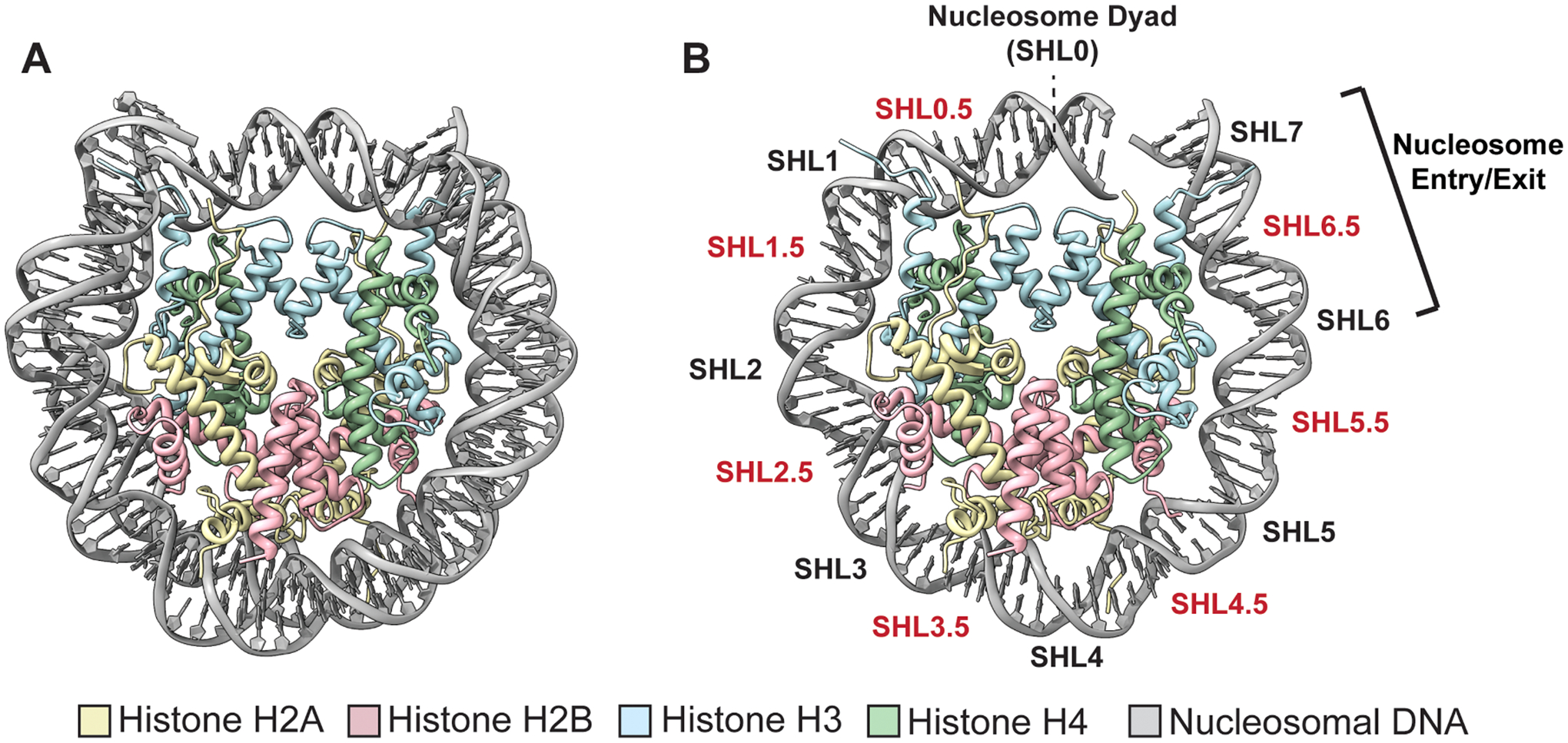
**(A)** Structure of a nucleosome core particle (adapted from PDB: 9DWF). **(B)** Structure of one half of the nucleosomal DNA in the nucleosome. Key regions of the nucleosomal DNA including the nucleosome dyad, nucleosome entry/exit site, and superhelical locations (SHLs) are labeled.

In eukaryotic cells, genomic DNA is packaged into chromatin through a fundamental repeating unit known as the nucleosome [2,3]. The nucleosome consists of ~147 bp of DNA wrapped in a left-handed superhelix around a core histone octamer composed of two copies each of histones H2A, H2B, H3, and H4 (Fig. 1 A) [4,5]. The nucleosome exhibits two-fold pseudo symmetry that is centered on the nucleosome dyad, also known as superhelical location 0 (SHL0), where the major groove of the DNA faces towards the core histone octamer. The nucleosomal DNA extends out ~73 bp in either direction towards the nucleosome entry/exit sites, and each additional inward facing major groove (every ~10 bp) is referred to as SHL ± 1–7. Importantly, the core histone octamer makes extensive contacts with the DNA throughout the nucleosome, which occurs when the minor groove faces the histone octamer at SHLs ± 0.5–6.5 (Fig. 1B). These oscillating contacts between the histone octamer and nucleosomal DNA ultimately creates a pattern of solvent-exposed and histone-occluded DNA regions that modulate nucleosomal DNA accessibility. While this packaging is essential for genome organization and regulation, it simultaneously restricts access to the genomic DNA, generating a physical barrier to essential nuclear processes including transcription, DNA replication, and DNA repair.

**Fig. 2.**
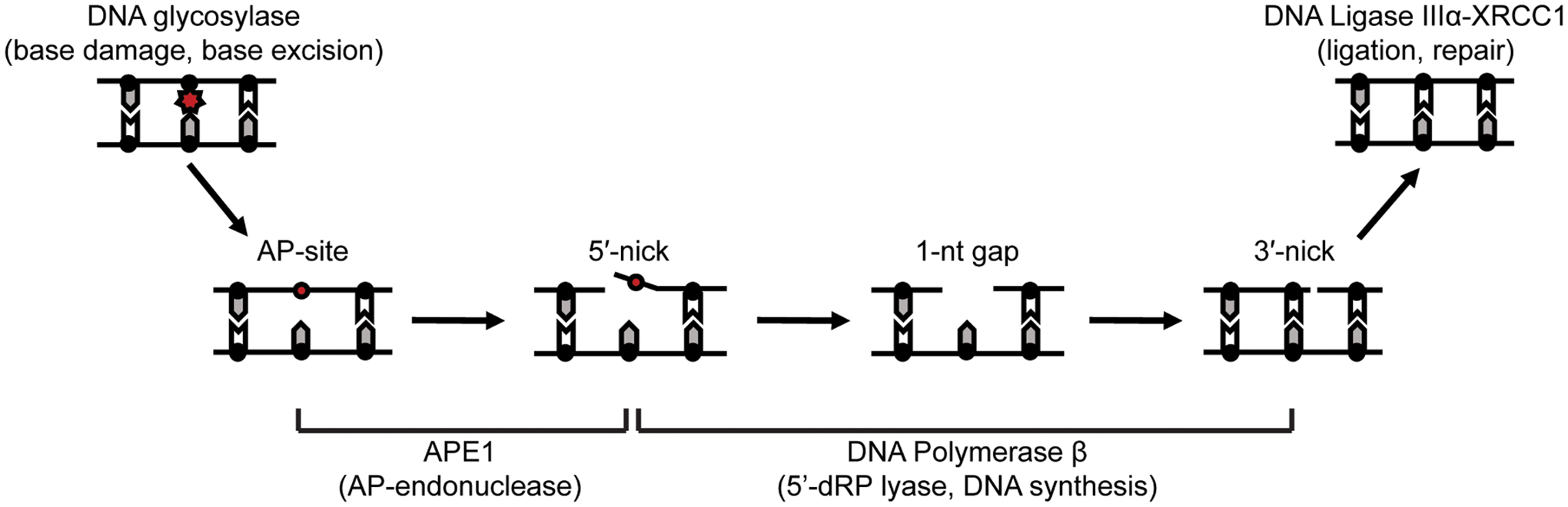
Schematic of the base excision repair (BER) pathway highlighting the core BER enzymes responsible for processing DNA base damage and BER intermediates (adapted from [1]).

The ubiquitous formation of DNA damage throughout the genome implies that DNA repair pathways must contend not only with a chemically diverse set of DNA lesions, but also with the physical constraints imposed by nucleosome structure and higher-order chromatin organization in order to maintain genome stability [6–13]. These challenges are particularly relevant for genome surveillance pathways like base excision repair (BER) (Fig. 2), which must efficiently access and process non-helix distorting DNA base lesions throughout the genome that arise from alkylation, oxidation, and deamination events [14–17]. BER is initiated by one of eleven lesion-specific DNA glycosylases that excise the damaged base, generating an apurinic/apyrimidinic (AP) site. AP endonuclease 1 (APE1) then incises the DNA backbone 5’ to the AP-site, creating a nicked intermediate bearing a 5’-deoxyribose phosphate (dRP) moiety. DNA polymerase β (DNA Pol β) subsequently excises the 5’-dRP and inserts an undamaged nucleotide in the place of the previously damaged base. BER is then completed by the X-ray repair cross-complementing protein 1 (XRCC1) and DNA ligase III alpha (LigIIIα) complex, which seals the remnant nick to restore DNA integrity. Of note, this description and the schematic in Fig. 2 reflects BER initiated by monofunctional glycosylases. Historically, the mechanistic description of how BER enzymes function have been derived from biochemical and structural studies using non-nucleosomal DNA substrates [18–20]. However, the physical constraints imposed by the nucleosome and higher-order chromatin add an additional layer of regulatory complexity. Fully elucidating the mechanisms of BER *in vivo* requires an understanding for how the core BER enzymes (i.e., DNA glycosylases, APE1, DNA Pol β, XRCC1/LigIIIα complex) recognize and repair DNA lesions within chromatin.

## 2. Overview of base excision repair in chromatin

The relationship between BER and chromatin in eukaryotes has been extensively studied over the past 50 years, and we refer readers to several comprehensive reviews on this topic [21–28]. Two foundational principles have emerged that shape our current understanding of BER in chromatin. First, DNA base lesions (e.g., oxidative and alkylative base lesions) form uniformly throughout chromatin structure, indicating BER must operate within chromatinized DNA. Second, BER efficiency is non-uniform in chromatin, where lesions in nucleosome-depleted regions of the genome are repaired more efficiently than those in nucleosome-dense regions. This differential repair efficiency correlates with elevated mutation rates in regions of chromatin where unrepaired oxidation- and alkylation-induced DNA base lesions persist, underscoring the biological importance of chromatin structure in modulating BER efficiency and mutagenesis [11–13].

Initial mechanistic insights into the non-uniform repair of DNA base lesions in chromatin came from numerous studies characterizing the enzymatic activities of core BER enzymes, including multiple DNA glycosylases [29–48], APE1 [29–33,38,41,49–53], DNA Pol β [29–33,38,44,54–60], and XRCC1-LigIIIα [54,61] on recombinant nucleosomes containing their cognate DNA lesions and/or repair intermediates. These investigations defined the kinetic basis of BER within nucleosomes *in vitro* (reviewed here [22,28,62–65]) and revealed several consistent themes that govern the enzymatic activity of BER enzymes in chromatin. First, core BER enzymes generally exhibit a reduced activity on nucleosomal DNA compared to non-nucleosomal DNA. Second, BER enzyme activity within the nucleosome is non-uniform (i.e., position dependent), which is largely dictated by the translational position (i.e. relative to the nucleosome dyad) and rotational orientation (i.e. relative to the histone octamer) of the DNA lesion (Fig. 1). For example, DNA lesions with translational positions near the nucleosome entry/exit site are processed more efficiently than those near the nucleosome dyad. In addition, DNA lesions with solvent-exposed rotational orientations are processed more efficiently than those with histone-occluded rotational orientations. These *in vitro* findings are consistent with genome-wide repair profiles of alkylative and oxidative DNA base lesions in cells, although this position-dependent repair is more pronounced *in vitro*, likely due to the absence of regulatory factors that modulate chromatin structure *in vivo* (see Section 3). While these biochemical studies revealed fundamental principles of BER efficiency within the nucleosome and chromatin, the structural mechanisms underlying position-dependent lesion processing remained unclear until recent cryo-EM studies began to elucidate how BER enzymes recognize and process DNA lesions within nucleosomes [13,53,60,66–68].

## 3. DNA sculpting drives lesion recognition in the nucleosome by core BER enzymes

In this section, we highlight recent findings that describe how the core BER machinery recognize and process DNA base lesions and repair intermediates within the nucleosome, emphasizing how structural insights explain the position-dependent enzymatic activity of BER enzymes on nucleosomal DNA. From this work, two key themes have emerged: (1) when the phosphate backbone is intact (e.g., base damage and AP-sites), BER enzymes employ a localized DNA sculpting mechanism for lesion recognition; and (2) when the phosphate backbone is broken (e.g., gaps and nicks), BER enzymes use a global DNA sculpting mechanism for lesion recognition.

### 3.1. Local DNA sculpting drives DNA base damage and AP-site recognition in the nucleosome

BER is initiated by a damage-specific DNA glycosylase that recognizes and excises a single damaged DNA base. Alkyladenine DNA glycosylase (AAG) and 8-oxoguanine glycosylase 1 (OGG1) are two enzymes responsible for initiating BER of alkylative and oxidative base damage, respectively. AAG excises alkylated or deaminated purine residues [69,70], whereas OGG1 excises 8-oxoguanine (8oxoG) opposite cytosine [71], with both enzymes generating an AP-site that is subsequently processed by APE1. BER can also be directly initiated by APE1, which incises AP-sites formed spontaneously via depurination or depyrimidination events [72,73]. A common theme for the initiation of BER is that DNA glycosylases and APE1 both act on DNA lesions containing an intact phosphate backbone, a defining feature that drives how they recognize DNA lesions within chromatin.

**Fig. 3.**
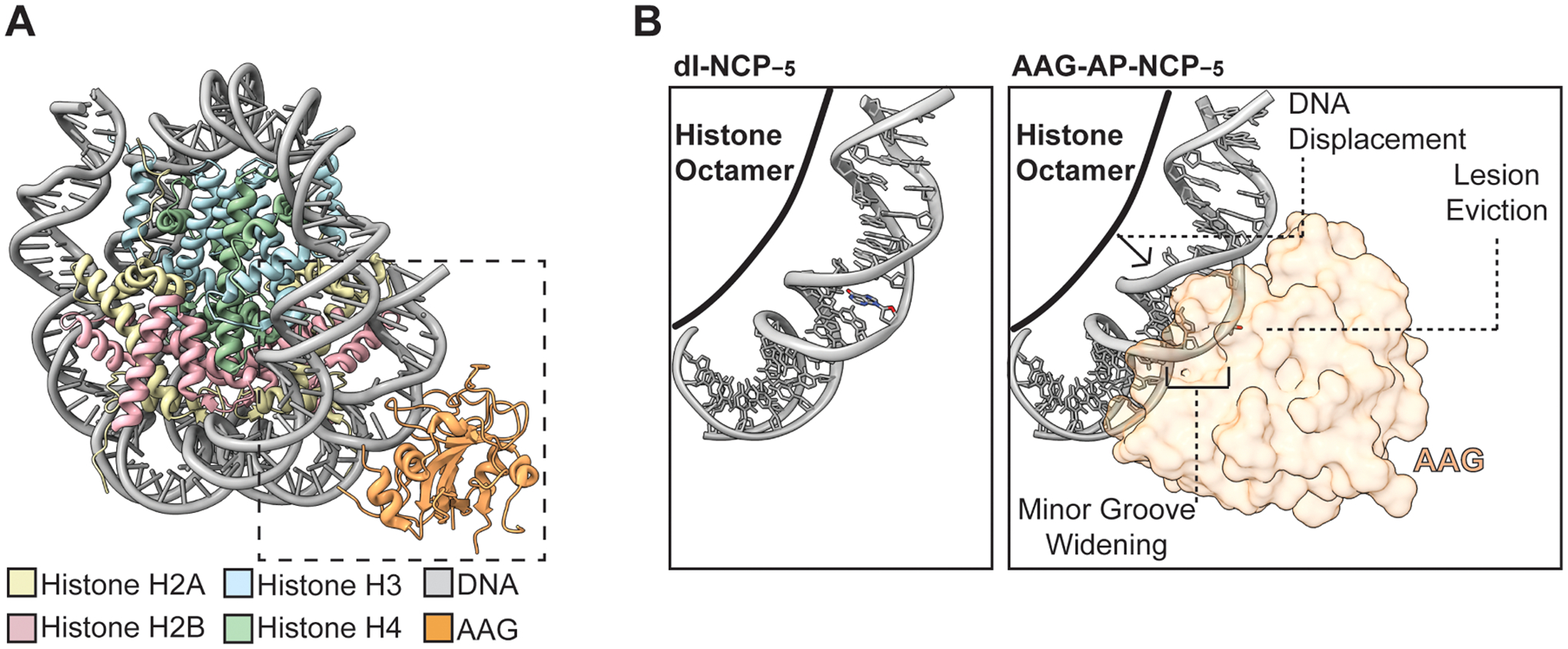
Structural basis of lesion recognition in the nucleosome by AAG. **(A)** Cryo-EM model of the AAG-AP-NCP–5 complex (PDB: 7XFJ). **(B)** Focused views of the nucleosomal DNA from SHL–4 to SHL–5.5 in the dI-NCP–5 (left) and AAG-AP-NCP–5 complex (right). The local deformations in the nucleosomal DNA induced by AAG during lesion recognition are labeled.

The first structural insight into DNA base damage recognition and excision in the nucleosome by a DNA glycosylase was reported in 2023, when Zheng *et al*. determined a series of cryo-EM structures of AAG bound to nucleosomes containing a solvent-exposed deoxyinosine (dI) lesion at SHL–5 and SHL–3, as well as a nucleosome containing a partially histone-occluded dI lesion at SHL–5.5 [66]. Notably, these structures were captured post-catalysis, after the dI had been converted to an AP-site, resulting in structures showing AAG bound to an AP-site. The structures revealed that AAG engages the AP-site and surrounding ~10 bp of DNA in a manner similar to its binding mode in non-nucleosome DNA (Fig. 3A) [74]. Recognition of solvent-exposed dI at SHL–5 and SHL–3 is facilitated by localized deformations in the nucleosomal DNA (i.e., localized DNA sculpting), including displacement of the DNA away from the histone core and widening of the minor groove. These distortions enable AAG to flip the DNA lesion from within the helix into its active site for catalysis, without steric conflict with the histone octamer (Fig. 3B). This lack of contact with the histone octamer likely explains the comparable activity of AAG on solvent-exposed nucleosomal lesions compared to non-nucleosomal lesions [46,75]. The structure of AAG bound to an AP-site with an intermediate level of solvent exposure at SHL–5.5 revealed that its lesion recognition, DNA binding, and local DNA sculpting mechanism closely resemble that for the solvent-exposed positions at SHL–5 and SHL–3. However, AAG repositions the partially occluded lesion at SHL–5.5 into the more solvent-exposed rotational setting at SHL–5, a mechanism previously termed register shifting [76]. This initial repositioning to a solvent-exposed rotational orientation allows AAG to access the AP-site using localized DNA sculpting (DNA displacement, bending, minor groove widening, and base flipping) without clashing with the histone octamer. Interestingly, register shifting requires the disruption and reformation of histone DNA contacts near the DNA lesion, which likely accounts for the lower excision efficiency of AAG on histone-occluded versus solvent-exposed lesions in the nucleosome [46,75]. Consistent with this, attempts at complex formation between AAG and nucleosomes containing DNA lesions at fully histone-occluded rotational orientations were unsuccessful [66], indicating that AAG cannot efficiently register shift these lesions to a solvent-exposed rotational orientation.

**Fig. 4.**
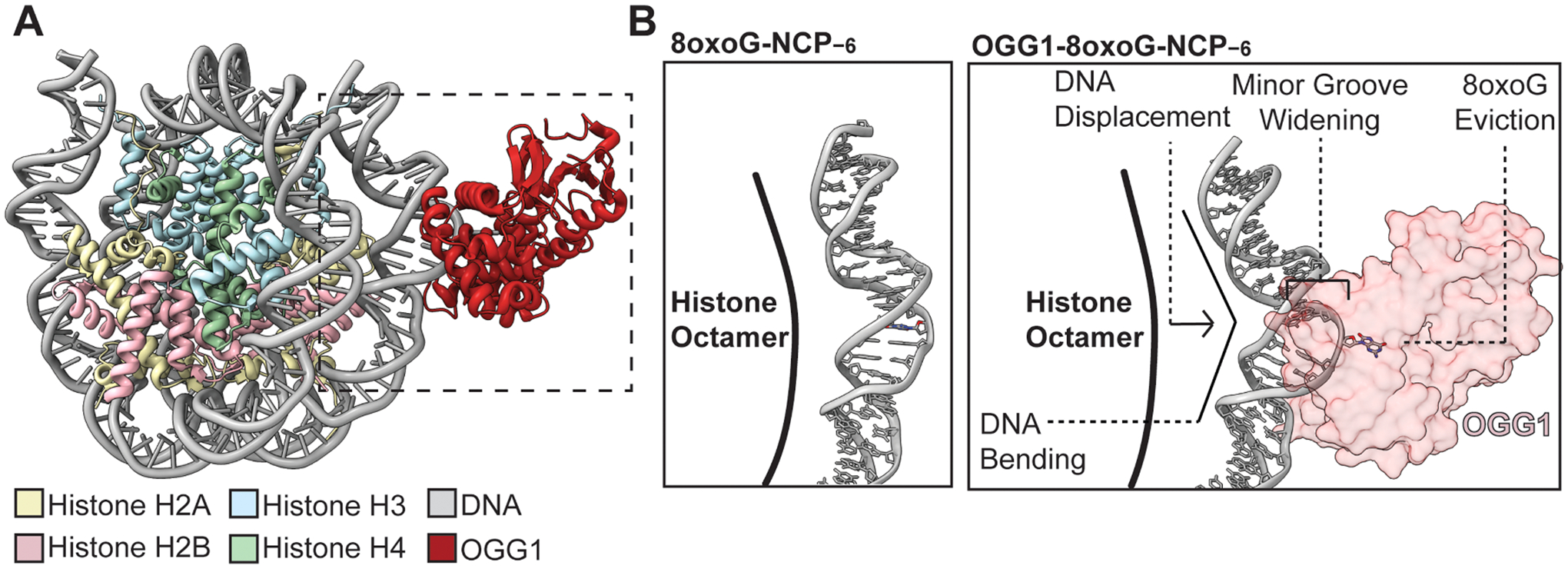
Structural basis of 8oxoG recognition in the nucleosome by OGG1. **(A)** Cryo-EM model of the OGG1–8oxoG-NCP–6 complex (PDB: 8VWT). **(B)** Focused views of the nucleosomal DNA from SHL–5 to the nucleosome entry/exit site in the 8oxoG-NCP–6 (left) and OGG1–8oxoG-NCP–6 complex (right). The local deformations in the nucleosomal DNA induced by OGG1 during 8oxoG recognition are labeled.

To further investigate how DNA glycosylases recognize and excise DNA base damage in the nucleosome, our group and others recently determined a series of cryo-EM structures of OGG1 bound to nucleosomes containing solvent-exposed 8oxoG at SHL–6, SHL+ 6, and SHL+ 4 [13,67]. These structures revealed that OGG1 engages the nucleosome through direct contact with the 8oxoG lesion and the surrounding ~6 bp of nucleosomal DNA (Fig. 4A), similar to its recognition mode in non-nucleosomal DNA [77]. Lesion recognition is mediated by local deformations in the nucleosomal DNA (i.e., displacement, bending, minor groove widening) that allow OGG1 to flip the 8oxoG from the DNA helix into its active site (Fig. 4B), reminiscent of the localized DNA sculpting mechanism observed for AAG. The similar mode of 8oxoG recognition at SHL–6, SHL+ 6, and SHL+ 4 suggests that OGG1 uses a common mechanism to engage solvent-exposed 8oxoG at different translational positions within the nucleosome. This approach enables OGG1 to access solvent-exposed lesions without clashing with the histone octamer, likely explaining its comparable catalytic activity on solvent-exposed lesions in the nucleosome and lesions in non-nucleosomal DNA [42,78]. Although structural insight into how OGG1 engages histone-occluded 8oxoG is lacking, it is plausible that OGG1 employs a register-shifting mechanism similar to AAG [66], which would explain why OGG1 has reduced efficiency on histone occluded lesions relative to solvent-exposed lesions in the nucleosome [39,42,78].

**Fig. 5.**
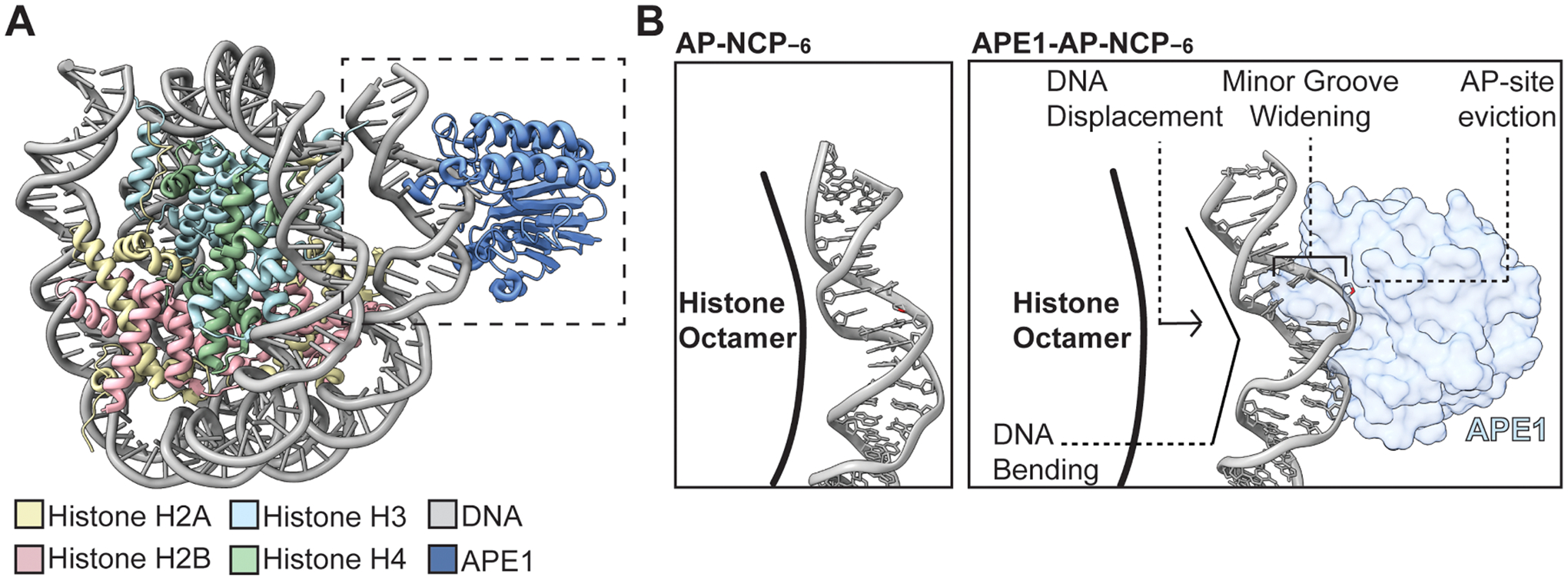
Structural basis of AP-site recognition in the nucleosome by APE1. **(A)** Cryo-EM model of the APE1-AP-NCP–6 complex (PDB: 7U50). **(B)** Focused views of the nucleosomal DNA from SHL–5.5 to the nucleosome entry/exit site in the AP-NCP–6 (left) and APE1-AP-NCP–6 complex (right). The local deformations in the nucleosomal DNA induced by APE1 during AP-site recognition are labeled.

APE1 is responsible for initiating BER at spontaneously formed AP-sites and processing AP-sites that arise as BER intermediates following base excision by DNA glycosylases. To investigate how APE1 recognizes and incises AP-sites in the nucleosome, our group recently determined a cryo-EM structure of APE1 bound to a nucleosome containing a solvent-exposed AP-site at SHL–6 [53]. This structure revealed that APE1 directly engages the lesion and the surrounding ~10 bp of nucleosomal DNA (Fig. 5A), similar to that observed for non-nucleosomal DNA [79,80]. Lesion recognition is mediated by local deformations of the nucleosomal DNA (i.e., displacement, bending, minor groove widening) that allow APE1 to flip the AP-site from the DNA helix into its active site without clashing with the histone octamer (Fig. 5B), reminiscent of the localized DNA sculpting observed for AAG and OGG1. This strategy allows APE1 to access solvent-exposed lesions without clashing with the histone octamer, likely explaining its comparable catalytic activity on solvent-exposed lesions in the nucleosome and lesions in non-nucleosomal DNA [29,30,32,38,49,50,53,59,81,82]. Additional structures of APE1 bound to AP-sites at other translational positions and rotational orientations will be needed to determine whether this mechanism is general across nucleosomal contexts, which would clarify the structural basis of position-dependent APE1 activity in the nucleosome.

Structures of AAG, OGG1, and APE1 bound to nucleosomes containing DNA lesions reveal a common strategy for lesion recognition when the DNA phosphate backbone is intact. For each of these enzymes, lesion recognition involves a set of local deformations in the nucleosomal DNA that are needed to reposition the DNA lesion into the enzyme active site (i.e., localized DNA sculpting), while simultaneously avoiding steric conflict with the histone octamer. This localized DNA sculpting mechanism enables these enzymes to efficiently process solvent-exposed lesions at multiple translational positions within the nucleosome. The same is not true for processing histone-occluded lesions in the nucleosome because the localized deformations in the nucleosomal DNA that are required for lesion recognition would result in substantial steric clashing between the repair enzymes and the histone octamer. Together, these findings suggest that localized DNA sculpting may be a shared mechanism of lesion recognition in the nucleosome during the initial steps of BER, though future structural studies of the remaining nine DNA glycosylases will be essential to determine whether localized DNA sculpting is always employed for lesion recognition in the nucleosome during the initiation of BER.

### 3.2. Global DNA sculpting drives single-strand break recognition in the nucleosome

The initial steps of BER give rise to a single-strand break intermediate (i.e., 5’-dRP) that is further processed by DNA Pol β and XRCC1-LigIIIα. DNA Pol β removes the 5′-dRP group and performs gap-filling DNA synthesis using two distinct active sites located in its N-terminal lyase domain (8-kDa domain) and C-terminal polymerase domain (31-kDa domain), respectively [20]. Following DNA Pol β activity, XRCC1-LigIIIα then seals the remnant nick to complete repair [83,84]. In contrast to DNA glycosylases and APE1, DNA Pol β and XRCC1-LigIIIα must process DNA lesions with a broken phosphate backbone, a defining feature that may drive how these enzymes recognize DNA lesions within chromatin.

**Fig. 6.**
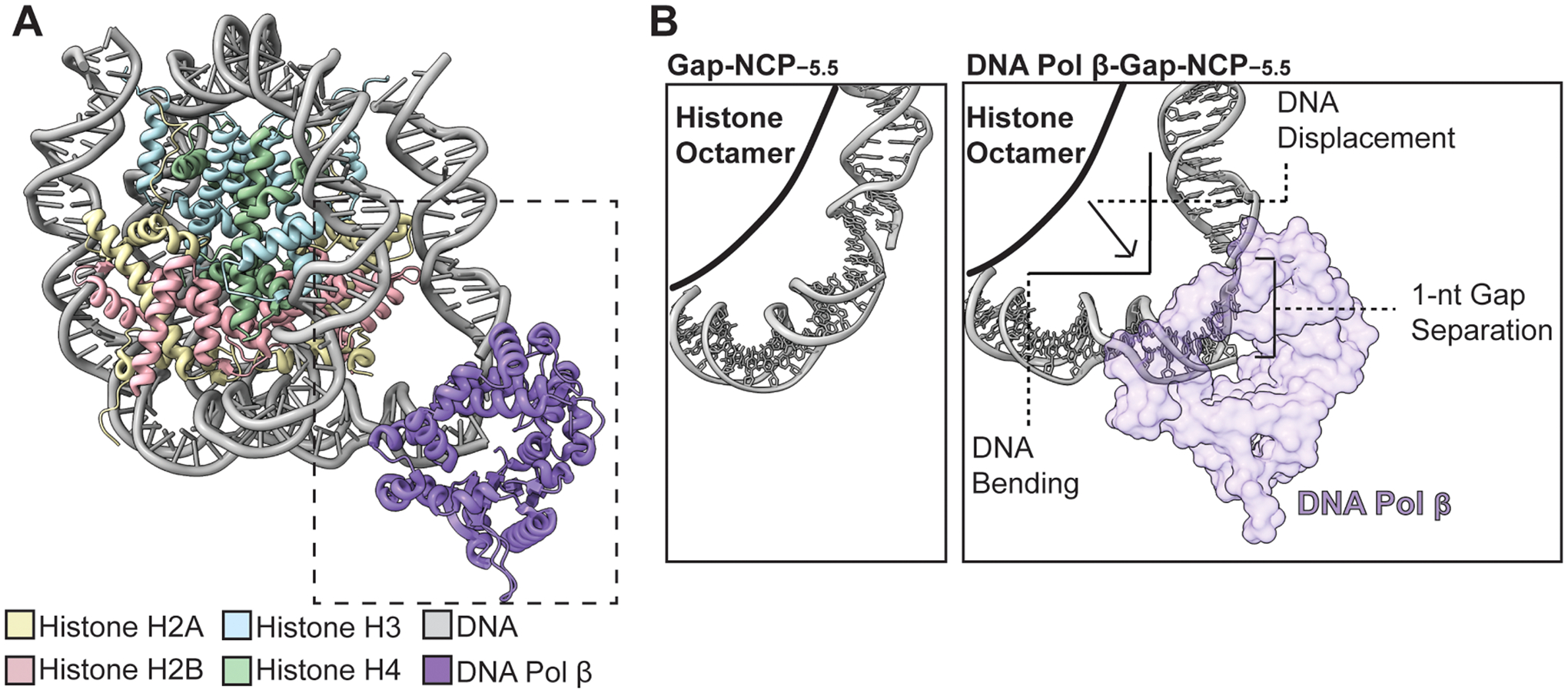
Structural basis of 1-nt gap recognition in the nucleosome by DNA Pol β. **(A)** Cryo-EM model of the DNA Pol β-Gap-NCP–5.5 complex (PDB: 9DWM). **(B)** Focused views of the nucleosomal DNA from SHL–3.5 to SHL–6 in the Gap-NCP–5.5 (left) and DNA Pol β-Gap-NCP–5.5 complex (right). The extensive deformations in the nucleosomal DNA induced by DNA Pol β during 1-nt gap recognition are labeled.

The first structural insight into how DNA Pol β recognizes 1-nt gaps and catalyzes nucleotide insertion in the nucleosome was reported in 2025, when our group determined a series of cryo-EM structures of Pol β bound to solvent-exposed 1-nt gaps in the nucleosome at SHL–5.5, SHL–4.5, and SHL–3.5 [60]. These structures revealed that nucleosome binding is mediated primarily by direct interaction with the 1-nt gap and the surrounding ~10 bp of nucleosomal DNA (Fig. 6A), similar to non-nucleosomal DNA [20,85,86]. Lesion recognition involves extensive deformations in ~35 bp of nucleosomal DNA (i.e., global DNA sculpting), including displacement away from the histone octamer, ~90° DNA bend, register shifting from the proximal entry/exit site, and inter-gyres widening (termed nucleosome gaping [87]) that separates the 5’-phosphate and primer terminal 3’-OH into the lyase and polymerase active sites, respectively (Fig. 6B). Notably, these structural deformations are far more extensive than those observed for AAG, OGG1, and APE1 during lesion recognition (Figs. 3–5). The substantial DNA deformations required for Pol β catalysis likely explain the reduction in nucleotide insertion rates for solvent-exposed 1-nt gaps in nucleosomal DNA compared to non-nucleosomal DNA [20,29,30,32,55–58,60]. Interestingly, one structure of Pol β bound to a 1-nt gap at SHL–3.5 lacks density for the terminal 21 bp of nucleosomal DNA at the proximal entry/exit site (i.e., unwrapping of the nucleosomal DNA), indicating that additional deformations in the nucleosomal DNA outside the Pol β binding footprint are required at less accessible translational positions in the nucleosome. These additional deformations in the nucleosomal DNA likely explains why Pol β catalyzes nucleotide insertion at solvent-exposed 1-nt gaps near the nucleosome dyad with substantially lower efficiency than at solvent-exposed gaps near the entry/exit site.

The 5’-dRP lyase activity of Pol β is the only known BER enzymatic activity that is minimally affected by nucleosome structure [58]. Although a structure of Pol β bound to a 5’-dRP in the nucleosome has yet to be determined, a structural intermediate with the lyase domain engaged with the 5’-phosphate during 1-nt gap recognition has provided initial insight into a possible recognition mechanism [60]. This structure revealed that binding of the 5’-phosphate, prior to engagement of the polymerase domain with the primer terminal 3’-OH, induces more modest deformations in the nucleosomal DNA. These modest deformations in the nucleosomal DNA may explain why the nucleosome has minimal impact on the 5’-dRP lyase activity of Pol β [58], in contrast to its polymerase activity [29–33,38,44,54–60], as recognition of the 5’-dRP would minimize structural rearrangements in the nucleosomal DNA. Nonetheless, structures of Pol β bound to a 5’-dRP intermediate in the nucleosome will be required to fully understand the basis of its robust 5’-dRP lyase activity, and to clarify how this step fits within the broader spectrum of BER enzyme catalytic activities within chromatin.

Following gap filling by Pol β, the final step of BER is catalyzed by XRCC1-LigIIIα, which seals the remaining nick in the DNA backbone [83,84]. Unfortunately, a structure of XRCC1-LigIIIα bound to a nucleosome containing a nick has yet to be determined. Nevertheless, the structural observations for DNA glycosylases [13,66,67], APE1 [53], and Pol β [60] indicate they use the same general mechanism to recognize DNA lesions in nucleosomal and non-nucleosomal DNA. This suggests that structures of LigIIIα bound to non-nucleosomal DNA containing a nick may provide some insight into nick recognition in the nucleosome. In non-nucleosomal DNA, LigIIIα encircles the duplex via its catalytic core and DNA-binding domains, inducing a sharp bend in the DNA near the nick site to align the 5′-phosphate and 3′-OH termini for ligation [88]. We envision that XRCC1-LigIIIα would also need to completely encircle and sharply bend the nucleosomal DNA during nick recognition. This would likely require a global DNA sculpting mechanism for lesion recognition like that observed for Pol β [60], rather than the localized DNA sculpting mechanism observed for DNA glycosylases and APE1 [13,53,66,67]. Future structural work will be needed to determine how XRCC1-LigIIIα recognizes nicks in the nucleosome to understand how BER is completed in chromatin.

Structures of Pol β bound to 1-nt gap containing nucleosomes reveal that global DNA sculpting entails large-scale distortions, including DNA displacement, bending, inter-gyre widening, register shifting, and in some cases DNA unwrapping, to gain access to DNA lesions. These distortions are facilitated in part by the broken DNA backbone of the 1-nt gap. While this mechanism enables Pol β to process solvent-exposed 1-nt gap BER intermediates, larger distortions are likely required when the 1-nt gap is in solvent-exposed positions at less accessible translational positions (e.g., near the dyad). Although structural data for XRCC1-LigIIIα bound to a nucleosome are lacking, its need to encircle and sharply bend DNA during nick recognition suggests that it may also employ a global DNA sculpting strategy. Collectively, these findings define global DNA sculpting as a mechanism that enables BER enzymes to access single-strand break intermediates in chromatin.

## 4. Regulation of BER by ATP-dependent chromatin remodeling enzymes

The position-dependent activity of core BER enzymes in the nucleosome suggests that many locations within the nucleosome are refractory towards successful BER *in vitro*. However, BER occurs with relatively high efficiency within cellular chromatin, implying that additional cellular factors play a role in facilitating access to these otherwise inaccessible nucleosomal DNA lesions. Consistent with this, various chromatin modifying-enzymes have been shown to directly or indirectly promote the repair of DNA base lesions and/or BER intermediates *in vivo* [40,89–100]. Among the most prominent types of chromatin modifying enzymes are ATP-dependent chromatin remodeling enzymes, which couple ATP hydrolysis to movement of the histone octamer relative to nucleosomal DNA, the exchange of histones and histone variants, and/or the disassembly of nucleosomes. A growing number of ATP-dependent chromatin remodeling enzymes have been identified that facilitate the repair of DNA base damage or BER intermediates in cellular chromatin [40,89–98,100]. Notably, many of these are recruited to damaged chromatin by Poly-(ADP-ribose) Polymerases (PARPs) and/or PARP-catalyzed histone ADP-ribosylation, directly linking them to the BER pathway [89,90,92–95,100–102]. Despite the strong link between ATP-dependent chromatin remodeling enzymes and BER, how these enzymes modify chromatin structure in response to DNA damage and how this impacts lesion recognition and repair within the nucleosome by core BER enzymes remains quite poorly understood.

The detailed structural analysis of core BER enzymes bound to DNA lesions in the nucleosome identified that local or global DNA sculpting is required for lesion recognition and repair in chromatin. It is likely that these ATP-dependent chromatin remodeling enzymes are responsible for generating a nucleosome and/or chromatin environment where local and global DNA sculpting by core BER enzymes is permissible. It is intuitive to speculate that these ATP-dependent chromatin remodeling enzymes move DNA damage from within the nucleosome to a location outside of the nucleosome to promote efficient BER. However, given that core BER enzymes are quite efficient at processing solvent-exposed lesions near the nucleosome entry/exit site, it’s possible that ATP-dependent chromatin remodeling enzymes are simply tasked with moving DNA lesions from inaccessible locations to more accessible locations within the nucleosome, such as repositioning DNA lesions from histone-occluded rotational orientations to solvent exposed rotational orientations or repositioning DNA lesions from translational positions near the nucleosome dyad to translational positions near the nucleosome entry/exit site. Indeed, we recently showed that the ATP-dependent chromatin remodeling enzyme ALC1 stimulates the enzymatic activity of the APE1 within the nucleosome without moving the AP-site outside of the core nucleosome footprint [93]. Finally, the possibility that multiple ATP-dependent chromatin remodeling enzymes work together or independently within certain chromatin contexts (e.g., euchromatin or heterochromatin) adds additional layers of complexity to this regulation. Future work will be needed to validate the impact of each of these ATP-dependent chromatin remodeling enzymes on cellular BER efficiency, to determine how these enzymes modify chromatin structure to facilitate lesion recognition and repair by the core BER machinery, and to decipher how these enzymes work together to facilitate DNA repair within higher-order chromatin. Ultimately, disentangling this interplay between ATP-dependent chromatin remodeling enzymes and the core BER machinery will likely be major driver of the field moving forward.

## 5. Conclusions and future directions

In this perspective, we summarized our current understanding of BER in chromatin from a structural perspective, highlighting several seminal papers that have begun refining the structural basis of lesion recognition and repair in the nucleosome by the core BER machinery. These structures have identified DNA sculpting as a common mechanism for lesion recognition in the nucleosome, where enzymes acting on an intact backbone (e.g., AAG, OGG1, APE1) typically rely on localized DNA sculpting for lesion recognition, while those acting on single-stranded break intermediates (e.g., DNA Pol β) rely on global DNA sculpting for lesion recognition. We envision this dynamic process is similar to a molecular “tug-of-war,” where the histone octamer and the BER enzymes are in a constant competition for access to the damaged nucleosomal DNA. The outcome of this competition is dictated by the position of the DNA lesion within the nucleosome, which ultimately defines the efficiency of BER enzymes within chromatin. From a cellular perspective, initial lesion recognition is a critical step in BER, and the ability to identify and engage lesions with minimal sculpting (e.g., AAG, OGG1, APE1) may enhance both the speed and fidelity of damage detection, effectively flagging compromised DNA for repair by BER. Once BER is initiated, it has been proposed that some BER enzymes can work in a coordinated fashion to enhance efficiency and prevent accumulation of toxic DNA intermediates [103]. The structural data summarized here suggests that BER enzymes may induce progressively greater DNA deformations in the nucleosome as the pathway advances. This raises the interesting possibility that downstream enzymes exploit pre-sculpted DNA configurations established by prior enzymes in the BER pathway.

Despite the advances summarized here, important opened-ended questions remain: (1) Do core BER enzymes assemble into multi-protein complexes on the nucleosome and what impact does co-complex formation on nucleosomes have on BER efficiency?; (2) How does higher-order chromatin architecture impact the DNA sculpting mechanism used for lesion recognition and repair by core BER enzymes?; and (3) How do ATP-dependent chromatin remodeling enzymes regulate chromatin structure to facilitate BER efficiency? Ultimately, future structural, biochemical, and cellular studies addressing these pressing unanswered questions will undoubtedly enhance our understanding for how BER occurs within the context of chromatin.
